# Pregnancy outcomes in nulliparous women after ultrasound ablation of uterine fibroids: A single-central retrospective study

**DOI:** 10.1038/s41598-017-04319-y

**Published:** 2017-06-21

**Authors:** Jun shu Li, Yong Wang, Jin yun Chen, Wen zhi Chen

**Affiliations:** 1 The State Key Laboratory of Ultrasound Engineering in Medicine Co-Founded by Chongqing and the Ministry of Science and Technology, Chongqing Key Laboratory of Biomedical Engineering, College of Biomedical Engineering, Chongqing Medical University, Chongqing Collaborative Innovation Center for Minimally-invasive and Noninvasive Medicine, Chongqing, 400016 China; 2 HIFU Center for Tumor Therapy, 1st Affiliated Hospital of Chongqing Medical University, Chongqing, 400042 China; 3 Clinical Center for Tumor Therapy, the Second Affiliated Hospital of Chongqing Medical University, 74 Lin jiang Road, Chongqing, 400010 China

## Abstract

To explore the impact of high-intensity focused ultrasound (HIFU) ablation of uterine fibroids in nulliparous women on subsequent pregnancy and delivery. A retrospective analysis was conducted of nulliparous women who received HIFU treatment at Chongqing Medical University, Chongqing,China, from January 1, 2010, to January 1, 2015. A total of 189 cases were enrolled, the median follow-up time was three years. Among them,there were 131 cases pregnancy with a total of 133 times,the pregnancy rate was 69.3% (131/189),and the spontaneous conception rate was 95.4% (125/131). Of 131 pregnant women, 19 were on-going pregnancy, terminated pregnancy 114 times,which include 93 times successfully delivery with a 76.3% (87/114) full-term birth rate,and the cesarean section rate was 72.0% (67/93). Of 94 newborns,the average birth weight was (3.3 ± 0.4)kg (range:1.5–4.8 kg), and a pair of them were identical twins. The incidence of complications during pregnancy and delivery were 10.8% (10/93) and 7.5% (7/93),respectively,except one woman failed on-going pregnancy and one woman suffered hysterectomy due to the complications,others all successful pregnant and delivered. Multiple-factor regression analysis found that age and infertility history were the important factors that may affect pregnancy after HIFU (P < 0.01). Nulliparous women who undergo HIFU treatment for uterine fibroids can subsequently have successful pregnancy and delivery safely.

## Introduction

Uterine fibroids are hormone-sensitive, benign tumors that are present in between 20% and 50% of women of reproductive age^1–3^, and the rate of pregnancy with uterine fibroids is between 2.7% and 20%^4–7^. Patients often present to the hospital with symptoms related to menstruation, or as a consequence of tumor mass effects on nearby organs. In addition, the presence of uterine fibroids alters the form and environment of the uterine cavity. These changes to the uterine cavity impact women of childbearing age by increasing the risk of infertility and miscarriage, as well as reducing the success rate of assisted reproductive blastocyst transplant^8–13^. As a result, treatment options for patients with uterine fibroids who wish to maintain fertility is a clinical challenge. Although myomectomy is recommended for patients who wish to maintain their fertility, it is not ideal due to the prolonged post-operation recovery time.

Furthermore, incision site pregnancy, iatrogenic placenta previa, and pelvic adhesions may increase the risk of infertility^14–16^. In patients with uterine scar tissue, there is a significantly increased risk of uterine rupture (the incidence of uterine rupture is 0.24–10.0%)^17–24^. The development of non-invasive treatment options has been increasingly focused upon in recent years. Although uterine artery embolization (UAE) can significantly reduce fibroid volume and improve symptoms^25–27^, there is a risk of ovarian failure that should be considered carefully in women who wish to maintain fertility^28–32^. Thus, there is still controversy regarding the use of UAE treatment of uterine fibroids in women who wish to maintain fertility.

High-intensity focused ultrasound (HIFU) is a noninvasive thermal ablation treatment that uses continuous magnetic resonance imaging (MRI) or ultrasound imaging throughout the procedure. This treatment has potential to shrink the volume of the fibroid, restore the shape of the uterus, and minimize symptoms without damaging the surrounding tissues. The safety and efficacy of HIFU in the treatment of benign uterine tumors has been confirmed in multiple clinical studies^33–35^, and HIFU has been recommended as a treatment option to reduce the symptoms of uterine fibroids^36^. However, there is little data available about pregnancy outcomes after HIFU, although there have been reports that indicate pregnancy is safe one year after HIFU treatment^37^. The enrollment criteria set for early clinical investigations of HIFU generally required that patients were not intending to conceive in the near future. Here we have performed a retrospective analysis to evaluate the impact of HIFU treatment for uterine fibroids on pregnancy and pregnancy outcomes in women.

## Materials and Methods

### Study population

A retrospective analysis was conducted of nulliparous women with uterine fibroids who underwent HIFU ablation at the Clinical Center for Tumor Therapy, Chongqing Medical University, Chongqing, China, from January 1, 2010, to January 1, 2015. Inclusion criteria for analysis were as follows: (1) aged 20–42 years. (2) desire to maintain fertility. (3) have normal sexual life without contraception after HIFU. Exclusion criteria were: underwent re-interventions including hysterectomy or bilateral oophorectomy after HIFU. The study was approved by the Ethics Committee at Chongqing Medical University. All methods were carried out in accordance with the approved ethical guidelines. Written informed consent was obtained from each patient before every procedure.

### Ultrasound ablation and post-treatment instructions

**Figure 1:**
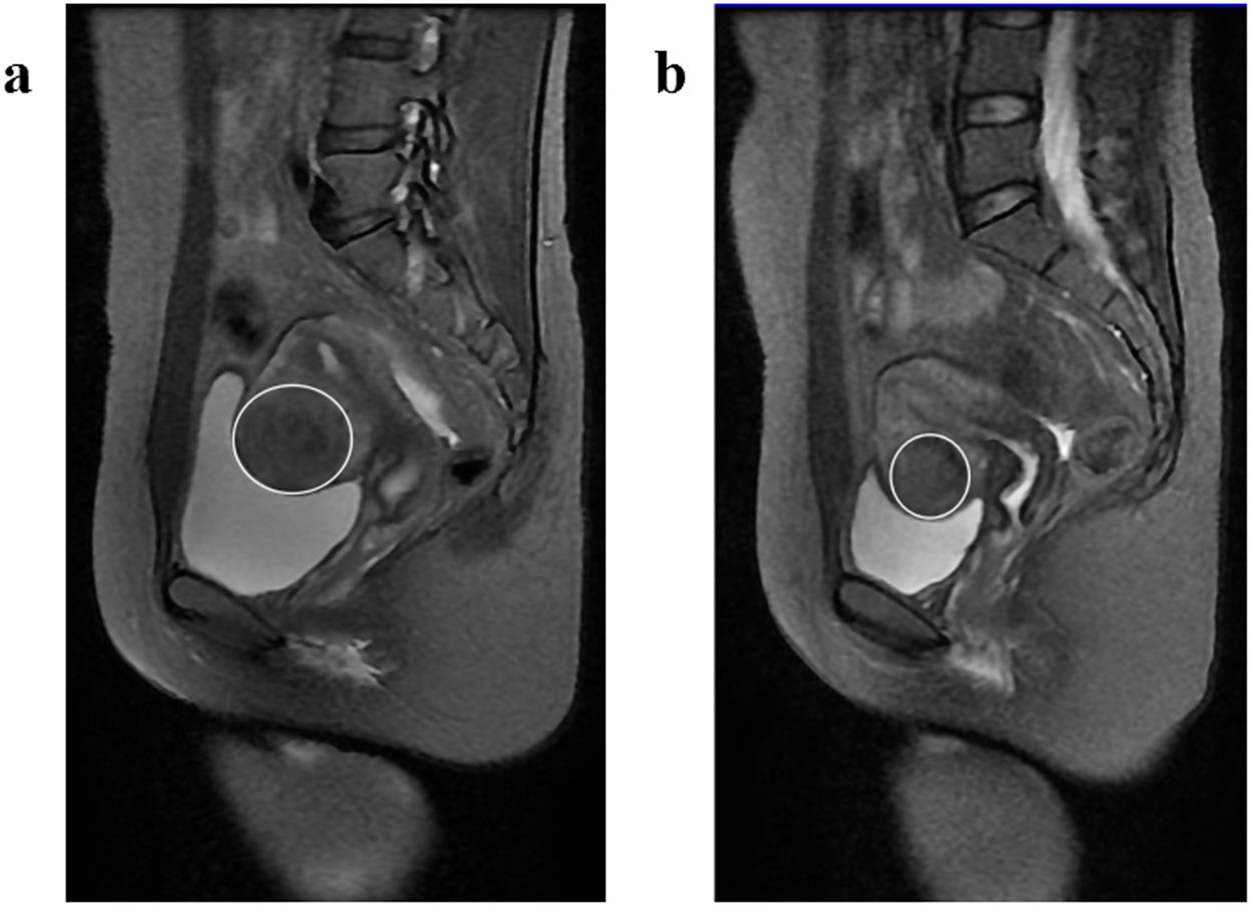
Magnetic resonance images showing the fibroid(white circles) before and after HIFU treatment. (**a**) before HIFU treatment, (**b**) three months after HIFU treatment.

Treatment was administered using the Model JC Focused Ultrasound Tumor Therapeutic System (Chongqing Haifu Medical Technology Co. Ltd.) with the microwave emission output power set to 200–400 W under continuous real-time ultrasound guidance at 3.5 MHz. The entire procedure was performed under conscious intravenous sedation with patients placed in the prone position. The effect of therapy was determined by color doppler ultrasound and contrast-enhanced ultrasound (SonoVue). Patients after HIFU should use contraceptives for 3 months and abstain from sex until after 1 menstruation. The recommendation for pregnancy planning was made after Magnetic resonance imaging (MRI) 3 months post HIFU treatment,for a representative case as a 31-year-old woman with uterine fibroid,on T2 weighted MR images showing the fibroid before HIFU and three months after HIFU treatment (Fig. 1). All the patients were advised to return for ultrasound examination at 3 months, 6 months, and 12 months, and then once a year thereafter. For some patients it was inconvenient to come to our hospital due to living a long distance away. These women were able to receive ultrasound examination in the local hospital where their uterine fibroids were diagnosed before HIFU treatment. The ultrasound examination report should be documented and sent to us by the network to be recorded for the study. The size of the myoma after HIFU were acquired from the ultrasound examination results. The diameter of myoma was measured in three perpendicular planes, with D1 as the longitudinal dimension, D2 as the anterior-posterior dimension, and D3 as the transverse dimension. The equation for calculating total fibroid volume used was V = 0.5233 × D1 × D2 × D3 (V is volume).

### Study Design

This is a single-center retrospective study. The data regarding marital status and birth history of patients who underwent HIFU ablation for uterine fibroids were screened through our electronic case record system. The inclusion criteria were women with uterine fibroids who never have delivery history before HIFU treatment. All of the nulliparous women were enrolled and called for follow up. An obstetrician and gynaecologist performed the telephone interviews with patients, and filled out the case follow-up form. Telephone follow-up information included menstruation, symptoms of uterine fibroids, sexual life, pregnancy, outcome of pregnancy, pregnancy process, and delivery information. The complications during pregnancy and delivery were acquired from the prenatal ultrasound examination report and medical discharge records.

Loss to follow-up was determined when the doctor failed to reach the patient by telephone after more than 3 attempts over the course of several days. We noted the number of patients who successfully completed telephone follow-up and those patients who were lost to follow-up.

### Statistical analyses

Statistical analyses were performed using SPSS 20.0 (SPSS Inc., Chicago, IL, USA). All data with a normal distribution are expressed as mean ± SD. Non-normally distributed data are expressed as median and range. Descriptive data such as age, body mass index, main fibroid volume, weight of newborn, interval between therapy and becoming pregnant, and complications during pregnancy and delivery were expressed as a range or rate. For analysis of related factors of postoperative pregnancy: in the single factor analysis (for preganacy related factors) classified variables were analyzed using Chi-square test or Fisher exact probability analysis, and disorderly classification variables were used for correlation analysis. Quantitative data were analyzed by cox regression. Multivariate regression analysis was performed for all variables with statistical significance in single factor analysis. Statistical significance was determined at P < 0.05.

## Results

### Patient Characteristics

**Figure 2:**
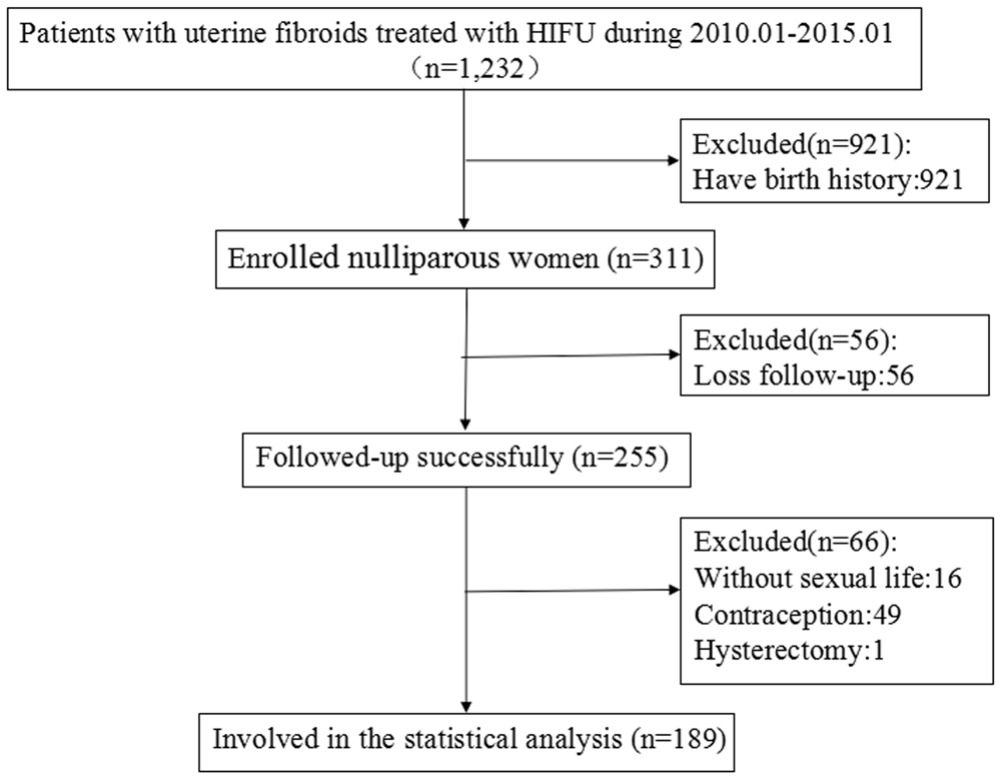
CONSORT diagram showing the disposition of patients in this analysis.

During the study period, there were 1,232 women that received HIFU treatment for uterine fibroids. Of these women, 311 patients met the inclusion criteria and follow-up data was obtained for 255 women. The median follow-up time was three years (range:1–5 years). Details regarding the excluded patients are shown in the flow chart (Fig. 2). Among those surveyed, 189 women reported having a normal sexual life without contraception after HIFU. At the time of treatment, the mean age of the 189 women was 31.4 ± 4.3 years (range, 23–42). Twenty of the women (10.6%) were more than 35 years old. Of the 189 women, 131 (69.3%) had a single fibroid and 58 (30.7%) had multiple fibroids when they were treated with HIFU. Up to the follow-up time, the symptom remission rates and average decrease of fibroid volume were 88.7% and 58.0 ± 31.3%, respectively (Table 1).

**Table 1:** Patient Characteristics of 189 women.

Variables	Value
Age (years)	31.4 ± 4.3 (23–42)
Median follow-up time (year)	3 (1–5)
Number of pregnancy loss (n)	1.0 ± 1.3 (0–7)
Infertility history (n, %)	45 (23.8)
Clinical symptoms before HIFU (n, %)*	159 (84.1)
Symptoms remission after HIFU (n, %)*	141 (88.7)
Number of myomas ablationed (n)	1.4 ± 0.9 (1–9)
Type of myoma^†^	
Intramural (n, %)	154 (81.5)
Subserous (n, %)	26 (13.8)
Submucous (n, %)	9 (4.8)
Main target myoma^†^	
Volume before HIFU (cm^3^)	81.2 ± 80.6 (4.4–505.2)
Volume after HIFU (cm^3^)	34.2 ± 48.3 (0–352.1)
Volume shrinkage (%)	58.0 ± 31.3 (0–100)

*Whether clinical symptoms were relieved according to the patient’s subjective evaluation. ^†^In cases of multiple myomas, these findings refer to only the largest myoma.

### Pregnancy and pregnancy outcome after HIFU

We identified 131 women who had a pregnancy after HIFU treatment. The total pregnancy rate was 69.3% (131/189). Within that, the rate of pregnancy in the first, second, third, fourth, and fifth year after HIFU intervention were 74.0% (97/131), 13.7% (18/131), 8.4% (11/131), 3.8% (5/131), 0.0%, respectively. The average age among the women who reported pregnancy was 30.3 ± 4.0 years (range: 23–40 years). The mean interval between HIFU treatment and pregnancy was 12.3 ± 9.9 months, The spontaneous pregnancy rate was 95.4% (125/131); the rate of pregnancy through assisted reproductive technology was 4.6% (6/131). The pregnancy rate of women with a history of infertility (infertility was defined as the inability to become pregnant for more than 12 consecutive months as reported by the patient) was 20.0% (9/45). Among the 131 women, 91 had successfully delivered, 19 were pregnant, four underwent abortion operation, and 17 pregnancies ended in spontaneous abortion at an average termination time of 2.2 ± 1.2 months (range:1–6 months) at the time of follow-up. Of those pregnancies that resulted in spontaneous abortion, 76.5% (13/17) had a previous history of pregnancy loss. Ninety-one women successfully delivered 93 times with a vaginal delivery rate of 28.0% (26/93), with the remainder delivering by cesarean section. Of those that delivered by cesarean section, 20.9% (14/67) chose cesarean section because of obstetric factors, 37.3% (25/67) for the fear of pain during labor, and the remaining 41.8% (28/67) for the advice of obstetrician (Table 2).

**Table 2:** Pregnancy outcomes after HIFU (n = 133).

Pregnancy outcomes	Cases (n)
Delivery mode	93 (70.0%)
Cesarean section*	67 (72%)
Vaginal delivery	26 (28%)
On-going pregnancy	19 (14.3%)
Abortion	21 (15.8%)
Spontaneous abortion	17 (12.8%)
Induced abortion	4 (3.0%)

*Including 6 cases (4.5%) termination pregnancy in premature for obstetric factors.

### Neonatal

Ninety-one women successfully delivered 94 newborns in total, two of the newborns were identical twins. Full term deliveries accounted for 93.6% (88/94) of the deliveries with 6.4% (6/94) of deliveries occurring preterm. Of the newborns, 52.1% were males and 47.9% were females. The average birth weight of all of the newborns was 3.3 ± 0.4 kg (range: 1.5–4.8 kg), four of them were low birth weight infants (defined as birth weight below 2.5 kilograms), two of the newborns exhibited macrosomia (Defined as birth weight exceed four kilograms), the rest were all healthy newborns (Table 3).

**Table 3:** Neonates characteristics (n = 94).

Variables	Value
Male/female (n)	49/45
Number of pregnancy (n)	93
Single pregnancy (n)	92 (98.9%)
Twin pregnancy (n)	1 (1.1%)
Pregnancy termination time (w)	37.5 ± 1.0 (33–39)
Birth weight (Kg)	3.3 ± 0.4 (1.5–4.8)
Macrosomia (n)	4.14/4.75
Low birth weight infants (n = 4)	
Premature infants (n = 2)	1.5/1.9
Full-term identical twins (n = 2)	2.45/2.25

### Complications during pregnancy and labor

In addition to the 19 cases of ongoing pregnancy and 21 cases of abortion, the remaining 91 cases in the 93 successful pregnancies and deliveries, the incidence rates of complications during pregnancy and labor were 10.8% (10/93) and 7.5% (7/93), respectively. Ten women had complications during pregnancy, including five with placenta previa, one with placental insufficiency, one with intrahepatic cholestasis, one with premature rupture of membrane, one with abnormal increase of ovarian cysts in the third trimester of pregnancy, and one with relaxation of internal orifice of uterus. In the five cases of placenta previa, three were marginal type,two were central type, and one each placenta increta among the two types of placenta previa. Of the ten cases that had complications during pregnancy, one woman had fetal intrauterine death at six months of gestation, which was due to the placental insufficiency and fetal growth restriction. The remaining nine women that experience complications in pregnancy continued pregnancy to at least 33 weeks of gestation resulting in six premature deliveries and three full-term deliveries. Of the 93 full-term deliveries, one case with intrauterine fetal distress occurred during labor and resulted in cesarean section. Finally, six cases with heavy hemorrhage during the third stage of labor, including one for complete placenta previa with placenta increta and one for uterine contraction inertia post-cesarean section, they were successfully rescued through hysterectomy and compression with suture incision to stop bleeding, respectively. The other four cases had heavy hemorrhage due to myomectomy during cesarean section, one case resulting in subtotal hysterectomy and the remaining three cases of hemorrhage were treated by compression with suture incision (Table 4).

**Table 4:** Outcomes and details of complications during pregnancy and labor.

Complications	Value (n)	Outcomes
Pregnancy complications (n, %)	10 (10.8%)	
Placenta previa	5	3 birth full-term, 2 birth premature
Placental insufficient	1	stillbirth
Intrahepatic cholestasis	1	Premature birth
Abnormal increase of ovarian cysts	1	Premature birth
Internal orifice of uterus relaxation	1	Premature birth
Premature rupture of membrane	1	Premature birth
Labor complications (n, %)	7 (7.5%)	
Fetal distress	1	cesarean delivery, full-term birth
Hemorrhoea due to central placenta	1	hysterectomy
Hemorrhoea due to uterine contraction inertia	1	Compression with suture uterus
Hemorrhoea due to myomectomy	4	1 subtotal hysterectom, 3 compression with suture uterus

### Comparative analysis clinical features of pregnancy after HIFU

Of the 189 women, the average age of the 131 pregnant women and the 58 non-pregnant women at the time of treatment were 30.4 ± 4.0 years and 33.8 ± 4.2 years, respectively, with the difference between the two groups being statistically significant (P < 0.01, P = 0.000). The number of women in the pregnancy group with a history of infertility was significantly lower than in the non-pregnancy group (6.9%, 9/131 vs 63.8%, 37/58, P < 0.01, P = 0.000). The continued remission of symptoms among women in the pregnancy group was significantly higher than that in the non-pregnancy group (98.2%, 108/110 vs 67.3%, 33/49, P < 0.01, P = 0.000). There were no significant differences in the type of myoma (P > 0.05, P = 0.860), volume of fibroid after HIFU (P > 0.05, P = 0.239), or the reduction of fibroid volume (P > 0.05, P = 0.094) between the two groups (Table 5). Multivariate regression analysis was performed for the above comparisons that were found to be statistically significant. Multivariate analysis indicated that age and a history of infertility were the factors that significantly affected pregnancy after treatment (P < 0.01, P = 0.009, P = 0.001).

**Table 5:** Characteristics between pregnancy group and non-pregnancy group.

Variables	Pregnancy group	Non-pregnancy group	P-values
Cases (n)	131	58	/
Age (years)	30.3 ± 4.0 (23–40)	33.8 ± 4.2 (25–42)	0.000
Infertility history (n, %)	9 (6.9)	36 (62.1)	0.000
Primary infertility (n, %)	5 (3.8)	25 (43.1)	
Secondary infertility (n, %)	4 (3.1)	11 (19.0)	
Type of myoma^†^			0.860
Intramural (n, %)	106 (80.9)	48 (82.8)	
Subserosal (n, %)	18 (13.7)	8 (13.8)	
Submucous (n, %)	7 (5.3)	2 (3.4)	
Number of myomas ablationed (n)	1.2 ± 0.8 (1–9)	1.6 ± 1.0 (1–5)	0.008
Clinical symptoms before HIFU (n, %)*	110 (84.0)	49 (84.5)	0.874
Symptoms remission after HIFU (n, %)*	108 (98.2)	33 (67.3)	0.000
Main target myoma^†^			
Volume before HIFU (cm^3^)	81.4 ± 81.5 (3.8–505.2)	80.1 ± 79.8 (4.4–372.0)	0.901
Volume after HIFU (cm^3^)	31.4 ± 43.1 (0.00–326.9)	40.4 ± 58.3 (0.0–352.1)	0.239
Volume shrinkage (%)	60.0 ± 30.8 (0–100)	52.3 ± 32.7 (0–100)	0.094

*Whether clinical symptoms were relieved according to patient’s subjective evaluation. ^†^In cases of multiple myomas, these findings refer to only the largest myoma.

## Discussion

HIFU is a type of noninvasive thermal ablation that can be used to treat uterine fibroids. Previous studies have found that, compared with laparoscopic myomectomy, HIFU has the advantages of fewer complications, faster recovery, less patient discomfort, and a low treatment-associated risk^38, 39^. All of this is in the context of the two treatments having similar efficacy, within the limitation that HIFU treatment is limited to ablation within the pseudomembrane. Because there is minimal damage to the surrounding normal myometrium, there is no obvious damage to the elastic and collagen fibers in uterine muscle after HIFU. Similarly, because there is less damage there is less scar tissue formation and less risk of collagen fiber hyperplasia. In turn, this theoretically would reduce the risks of pregnancy in women who have undergone HIFU treatment for uterine fibroids, as compared to myomectomy. Previous clinical studies have confirmed that HIFU does not impair ovarian function^37, 40^, does not further impede the ability to conceive, and does not increase the incidence of adverse reactions^41–43^.

In this study, we found that symptoms of uterine fibroids improve significantly after HIFU treatment, and the volume of the uterine fibroids decrease, (*P* < 0.05). Although we did not take into account the factor of male infertility, the pregnancy rate after HIFU still reached 69.3%, which is similar to the pregnancy rate (62.2–68%) after myomectomy^20, 44, 45^. Almost 74.0% of women had a pregnancy within one year after HIFU treatment. The spontaneous pregnancy rate after HIFU was 95.4%, which is slightly higher than that after myomectomy (64.6–88.6%)^19, 20^. Although the spontaneous abortion rate (14.9%) after HIFU is similar to that of after myomectomy (13.0–24%)^19, 20^, this rate is still significantly lower than in pregnancy with untreated fibroids present (20–46.7%)^13, 46^. Furthermore, 76.5% of the women who experienced spontaneous abortion had a prior history of pregnancy loss. There were 91 women who successfully delivered 93 times and give birth to a total of 94 healthy newborns. The results of this study indicate that women with uterine fibroids have a higher pregnancy rate and full-term delivery rate after HIFU treatment, as compared to treatment with myomectomy.

In terms of safety during pregnancy, we found that the incidence rate of pregnancy complications was 10.8%, similar to that of myomectomy (6.8–13%)^19, 20, 44, 47, 48^. The most common complications of pregnancy in this study were placenta previa and placenta implantation. Further, 83.3% of the complications occurred in patients with a past surgical history significant for uterine surgery. This may contribute to poor formation of uterine decidua blood vessels during pregnancy, which in turn increases the risk of insufficient placental blood supply. We believe this may explain our finding of a higher incidence of placental abnormalities than has been previously reported in the literature (5.4% vs 1.4%)^46^. It is difficult to speculate on whether there is an increased risk of placental abnormalities after HIFU treatment in this study, though this is a topic that warrants further investigation in the future. The incidence of premature delivery was significantly lower in those treated with HIFU than either myomectomy or pregnancy with untreated uterine fibroids (5.3% vs 10.0–10.3% vs 20–33.3%)^13, 19, 20, 46, 49^. This suggests that the risk of preterm birth is reduced after HIFU treatment. The source of this difference in outcomes may be an additional fruitful topic for further research.

In terms of labor safety, the most important delivery complications are postpartum hemorrhage, amniotic fluid embolism, and uterine rupture. The study presented here did not contain any cases of uterine rupture during pregnancy or labor after HIFU treatment. Previously published reports indicate a rate of uterine rupture during pregnancy and labor after myomectomy of 0.24–10%^17–24^. It is possible that the high proportion of deliveries by cesarean section in this group may influence this result. Consequently, the risk of uterine rupture is needs to be evaluated further with a large sample population. Similarly, although we only identified one case of fetal distress during vaginal delivery in our study sample set, which is lower than that of delivery under normal conditions (0.9% vs 2.5–10%)^50, 51^, this effect may also be attributed to the high rate of delivery by cesarean section in this study. In our study, the incidence rate of massive hemorrhage during labor and post-delivery was 6.5%. In addition to the factors of central placenta previa with placenta increta and uterine atony post- cesarean section, the majority (66.7%) were associated with resection of an intramural fibroid during cesarean section, which lead to one women undergoing a subtotal hysterectomy. Due to the rich blood supply around the uterus during the third trimester of pregnancy, myomectomy during cesarean section will increase bleeding. A previously published study suggested that it is safe to remove submucous and subserous leiomyomas during cesarean section, but the safety of removal of an intramural leiomyoma remains unknown^52^. Therefore, the removal of fibroids during cesarean section may be an important risk factor for the increase of labor complications, which should be taken into consideration by clinicians.

Many factors affect the decision of delivery method, including both obstetric and social factors. Because the large-scale application of HIFU for clinical treatment of uterine fibroids is recent to the last ten years, appropriate clinical data for evidence-based practices has been limited. Our findings indicate that the rate of cesarean section is similar to post-myomectomy (72.0% vs 66.7–78%)^19, 20, 53^. In our study, fourteen women (20.9%) chose delivery by cesarean section for obstetric factors, while 79.1% chose delivery by cesarean section for social factors. We believe that as clinical experience with HIFU increases and increasing data is evaluated, there will be a further reduction in delivery by cesarean section after HIFU.

There remain limitations to this study. First, because of our method of data collection and the retrospective nature of the study, we lack the ability to identify certain factors that may affect pregnancy and delivery after HIFU, such as male infertility. This will require a future prospective study with appropriate controls. Second, rare complications such as uterine rupture during pregnancy or labor were not identified in this study. However, whether this is due to HIFU or an effect of small sample size we cannot make any conclusions about the risk of these complications. Third,there were 28.6% (54/189) cases received follow-up ultrasound examination in the hospital near their home,where their uterine fibroid had been diagnosed before HIFU,it is possible that a different ultrasonographer may cause some measurement bias, which is the biggest drawback for a retrospective study. Future prospective study designs can address this by being more stringent for the data collection criteria. Finally, the age range of women in the study population is large. A future prospective study would be needed to address the potential influence of age on the outcomes described here.

In conclusion, HIFU is a safe and effective noninvasive therapy to treat uterine fibroids in nulliparous women who wish to retain the ability to conceive and deliver after treatment. Additionally, HIFU effectively provides remission of symptoms, has a low rate of complications through pregnancy and labor, and does not increase the rate of spontaneous abortion or delivery by cesarean section. Further, HIFU treatment has the advantages of being noninvasive, safe, effective, and a rapid recovery, which helps to minimize the risks associated with a surgical operation. Based on our findings presented here, we believe that HIFU should be the recommended treatment for uterine fibroids in women who wish to retain the ability to have children in the future, or who otherwise wish to not undergo hysterectomy.
